# Development of growth selection systems to isolate a-type or α-type of yeast cells spontaneously emerging from *MAT*a/α diploids

**DOI:** 10.1186/1754-1611-7-27

**Published:** 2013-11-21

**Authors:** Nobuo Fukuda, Shinya Honda

**Affiliations:** 1 Biomedical Research Institute, National Institute of Advanced Industrial Science and Technology (AIST), Higashi, Tsukuba, Ibaraki 305-8566, Japan

**Keywords:** Yeast, Biotechnology, Gene regulation, Transcription factors, Promoters

## Abstract

**Background:**

Manufacture of *MAT*a and *MAT*α yeast cells is required for crossbreeding, a procedure that permits hybridization and the generation of new heterozygous strains. Crossbreeding also can be performed with a- and α-type of cells, which have the same mating abilities as *MAT*a and *MAT*α haploid cells, respectively.

**Results:**

In this work, we describe a method to generate a- and α-type of cells via the naturally-occurring chromosomal aberration in parental *MAT*a/α diploids. We successfully designed suitable genetic circuits for expression of the *URA3* selection marker gene to permit isolation of a- and α-type of cells, respectively, on solid medium lacking uracil. Furthermore we succeeded in generation of zygotes by mating of both the manufactured a- and α-type of yeast cells.

**Conclusions:**

This process does not require exposure to mutagens such as UV irradiation, thereby avoiding the accumulation of undesirable mutations that would detract from the valuable traits that are under study. All the genetic modifications in the current study were introduced into yeast cells using plasmids, meaning that these traits can be removed without altering the genome sequence. This approach provides a reliable and versatile tool for scientific research and industrial yeast crossbreeding.

## Background

Crossbreeding is an effective approach used to improve and combine traits of yeast strains by zygosis of cells of opposite mating types (*MAT*a and *MAT*α) [1-3]. Generally, parental diploid and polyploid strains used for industrial application are never able to mate directly, and hence isolation of mating strains (typically, haploid strains) via sporulation is a prerequisite for crossbreeding. Yet classical crossbreeding can be problematic because numerous industrial yeast strains sporulate poorly or not at all [4-8].

The *MAT* genes locate on chromosome III that is the most unstable one among 16 chromosomes of *Saccharomyces cerevisiae*[9]. Naturally, a- and α-type of yeast cells emerge from *MAT*a/α diploids with quite low frequency, due to chromosomal aberration during mitotic division. These cells can be used as alternative mating strains, possessing the same mating ability as *MAT*a and *MAT*α haploids generated via sporulation, respectively.

There are several kinds of chromosomal aberration such as loss of heterozygosity (LOH) and mitotic chromosome loss. LOH is a natural event that generates homozygous loci via chromosomal rearrangement in heterozygous loci [10-13], and LOH occurring at the *MAT* locus within a *MAT*a/α cell produces either a *MAT*a/a or a *MAT*α/α cell. The spontaneous frequency of LOH is below 1 × 10^-4^[14]. Mitotic chromosome loss is also a naturally-occurring event that polyploid cells lose single or multiple chromosomes [15]. The frequency of loss of chromosome III in yeast diploid cells was reported to be 5 × 10^-5^[9], and yeast cells having lost one of two chromosomes III containing the *MAT*a or *MAT*α gene acquire either a- or α-type of mating ability. Unfortunately, however, it can be difficult to isolate the generated a- and α-type of cells, since the spontaneous frequencies of such events are quite low.

Ultraviolet (UV) irradiation of yeast diploid cells has been used successfully to increase the frequency of LOH to about 30% [16]. According to other report, chemicals such as benzonitrile and methyl ethyl ketone increase the frequency of chromosome loss [17]; however, these treatments have the potential to randomly induce undesirable mutations at loci other than the *MAT* locus or undesirable loss of chromosomes other than chromosome III, which might prevent the generated a- or α-type of cells from inheriting the desired properties of the parental strains when used in crossbreeding.

**Figure 1:**
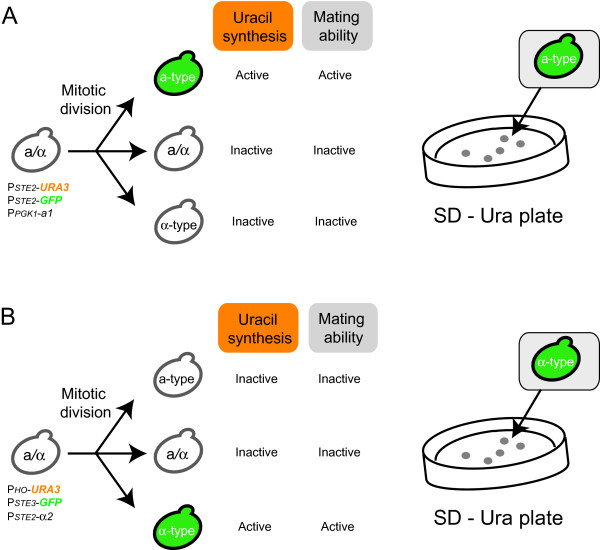
**Schematic outline of experimental design. (A)** Isolation of a-type of cells generated via spontaneous chromosomal aberration. *P*_*PGK1*_-a1 suppresses mating ability of α-type of cells to prevent autopolyploidization (mating between a- and α-type of cells derived from the same parental strain). The *URA3* gene is expressed only in a-type cells, and a-type of cells would be selected on SD solid medium lacking uracil (SD – Ura plates). **(B)** Isolation of α-type of cells generated via spontaneous chromosomal aberration. *P*_*STE2*_-α2 suppresses mating ability of a-type cells to prevent autopolyploidization. The *URA3* gene is expressed only in α-type cells, and α-type of cells would be selected on SD – Ura plates. The *GFP* reporter gene is used to detect yeast cells with target mating-type.

Here we designed genetic circuits to express the *URA3* gene as a selection marker, permitting isolation of the rare a- or α-type of cells arising via spontaneous chromosomal aberration (Figure 1). Furthermore, we introduced a previously constructed expression system to prevent autopolyploidization between a- and α-type of cells generated from the same parental strain. Specifically, we used the a1-α2 complex [18], a repressor of haploid-specific genes (*hsg*) that endow yeast cells with the ability to mate [19]. In this work, we show the feasibility of this approach and its potential to provide mating strains for yeast crossbreeding.

## Results

### General strategy

An outline of the experimental design for production of a- and α-type of cells is shown in Figure 1. Spontaneous chromosomal aberration during cultivation provide low numbers of both a- and α-type of cells in cultures containing large numbers of parental *MAT*a/α cells. The inclusion in our system of the a1-α2 complex (which represses expression of *hsg*) suppresses mating by the rare a- and α-type of cells, which therefore can grow independently in the cell mixture without forming non-hybrid (autopolyploid) cells [18]. Here we used *URA3* as the selection marker gene for a- (Figure 1A) or α-type of cells (Figure 1B), permitting isolation by selection on solid medium lacking uracil (SD – Ura plates).

All the genetic modifications in the current study were introduced into yeast cells using plasmids, permitting complete removal of the modification following isolation of the target cells. Final plasmid sets suitable for isolation of a- or α-type of cells were defined by sequential trial and error. The process is described below in a step-by-step manner.

### Trial 1: expression of the *URA3* gene via YEp-type plasmids

**Figure 2:**
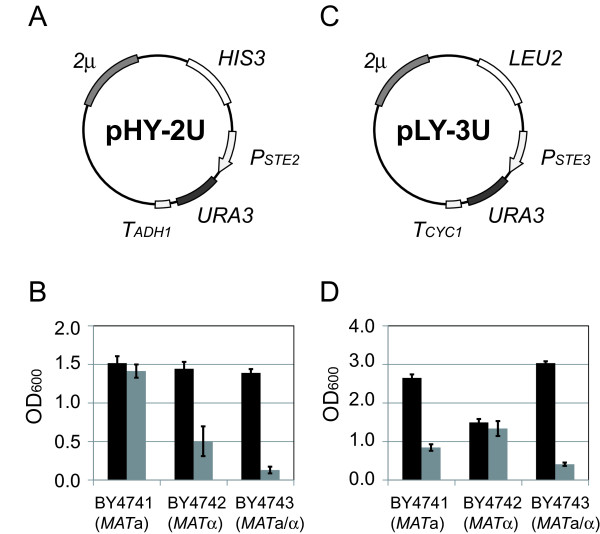
**Growth of yeast transformants harboring YEp-type plasmids. (A)** Plasmid map of pHY-2U containing *2 μ* origin of replication (providing cellular retention of multicopy plasmids) and *P*_*STE2*_-*URA3* construct (activated in a-type yeast cells). **(B)** The OD_600_ values of cultures of pHY-2U transformants at 18 h cultivation. Black bars indicate cultivation with uracil, and gray bars indicate cultivation without uracil. Values are presented as means ± standard deviations from three independent experiments. **(C)** Plasmid map of pLY-3U containing *2 μ* origin of replication and *P*_*STE3*_-*URA3* construct (activated in α-type yeast cells). **(D)** The OD_600_ values of cultures of pLY-3U transformants at 18 h cultivation. Symbols are as in B.

Two kinds of YEp-type (multi-copy) plasmids were constructed to express the *URA3* gene in a- or α-type of cells (Figure 2). Plasmid pHY-2U (Figure 2A) places the selection marker under control of *P*_*STE2*_, a promoter of a-type-specific genes (*asg*), so that only *MAT*a cells can express the *URA3* gene. On the other hand, plasmid pLY-3U (Figure 2C) places the selection marker under control of *P*_*STE3*_, a promoter of α-type-specific genes (*αsg*), so that only *MAT*α cells can express the *URA3* gene. Growth of each transformant was evaluated by monitoring the OD_600_.

Only *MAT*a cells possessing pHY-2U grew without uracil at the same level as with uracil (Figure 2B). Although there were obvious differences in growth ability between *MAT*a cells and others, *MAT*α and *MAT*a/α cells harboring pHY-2U exhibited some growth in the absence of uracil. As shown in Figure 2D, *MAT*a and *MAT*a/α cells harboring pLY-3U exhibited levels of growth equivalent to that of *MAT*α cells harboring pLY-3U when cultured on SD - Ura. These results suggest that leaky expression of the *URA3* gene via YEp-type of plasmids would preclude isolation of target cells on SD - Ura plates.

### Trial 2: expression of the *URA3* gene via YCp-type plasmids

**Figure 3:**
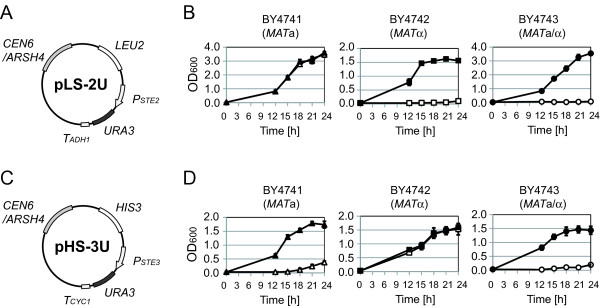
**Growth of yeast transformants harboring YCp-type of plasmids. (A)** Plasmid map of pLS-2U containing *CEN6/ARSH4* origin of replication (providing cellular retention of single-copy plasmids) and *P*_*STE2*_-*URA3* construct (activated in a-type yeast cells). **(B)** The growth curves of pLS-2U transformants. Closed symbols indicate cultivation with uracil, and open symbols indicate cultivation without uracil. Values are presented as means ± standard deviations from three independent experiments. **(C)** Plasmid map of pHS-3U containing *CEN6/ARSH4* origin of replication and *P*_*STE3*_-*URA3* contruct (activated in α-type yeast cells). **(D)** The growth curves of pHS-3U transformants. Symbols are as in B.

To reduce leaky *URA3* gene expression, DNA fragments containing *P*_*STE2*_-*URA3* or *P*_*STE3*_-*URA3* were transferred into YCp-type (single-copy) plasmids to yield pLS-2U (Figure 3A) or pHS-3U (Figure 3C), respectively. To make detailed examination of changes in growth ability of yeast cells, we tracked OD_600_ values over time, and then plotted growth curves for each transformant with or without uracil. Whereas pLS-2U permitted *MAT*a cells to grow without uracil, *MAT*α and *MAT*a/α cells harboring pLS-2U exhibited only minimal growth in the absence of uracil (Figure 3B). These results suggested that pLS-2U is suitable for isolation of a-type of cells.

Among cells transformed with pHS-3U, *MAT*a and *MAT*a/α grew poorly without uracil compared to *MAT*α (Figure 3D). Although the leakiness of *URA3* gene expression was significantly reduced by altering plasmid type from YEp to YCp, further optimization was needed to establish an isolation method for α-type of cells.

### Trial 3: use of the a1-α2 repressor complex combined with *P*_*HO*_, a promoter of *hsg*, for α-type-specific *URA3* gene expression

**Figure 4:**
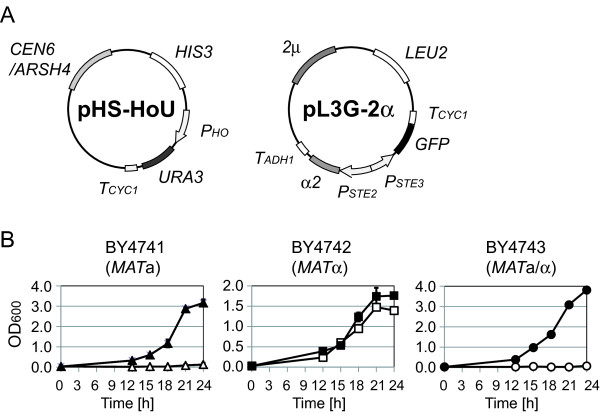
**Improved growth selection system for α-type of cells. (A)** Plasmids used for α-type-specific *URA3* gene expression. The plasmid pHS-HoU contains *CEN6/ARSH4* origin of replication (providing cellular retention of single-copy plasmids) and *P*_*HO*_-*URA3* construct (activated in yeast haploid cells). The plasmid pL3G-2α contains *P*_*STE2*_-α2, a construct that represses *URA3* gene expression in a-type yeast cells. **(B)** The growth curves of double plasmid (pHS-HoU and pL3G-2α) transformants. Closed symbols indicate cultivation with uracil, and open symbols indicate cultivation without uracil. Values are presented as means ± standard deviations from three independent experiments.

Two plasmids shown in Figure 4A were used in an improved strategy for α-type-specific *URA3* gene expression. The plasmid pL3G-2α that contains *P*_*STE2*_-α2 suppresses the mating ability of *MAT*a cells by artificially forming the a1-α2 repressor complex [18]. Because the a1-α2 complex can directly repress gene expression under the control of promoters of *hsg*, we selected *P*_*HO*_ (a *hsg* promoter) as an alternative promoter to express the *URA3* gene. Hence, as shown in Figure 1B, *P*_*HO*_-*URA3* is expected to be activated only in *MAT*α cells, where *P*_*STE2*_-α2 can drive expression off the *HO* promoter.

Figure 4B shows growth curves of transformants in the improved strategy. As expected, while *MAT*α transformants grew without uracil, *MAT*a and *MAT*a/α transformants exhibited minimal or no growth without uracil. These results suggest that use of pHS-HoU combined with pL3G-2α is suitable for isolation of α-type of cells.

### Artificial regulation network designed for isolation of a- and α-type of cells

**Figure 5:**
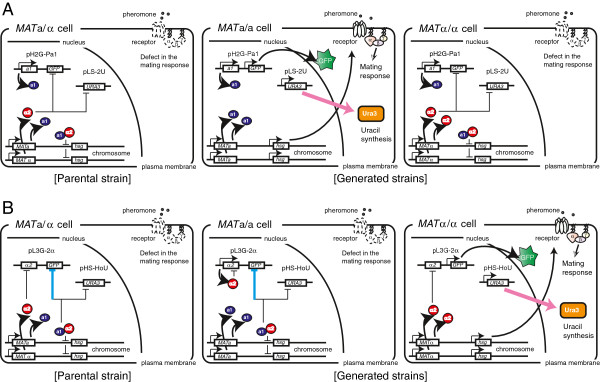
**Schematic outline of yeast mating-type regulation and our engineered approach. (A)** Engineering for isolation of a-type of cells. The *MAT*a/α parental strain indicates BY4743 harboring pH2G-Pa1 and pLS-2U (see Table 1). **(B)** Engineering for isolation of α-type of cells. The *MAT*a/α parental strain indicates BY4743 harboring pL3G-α2 and pHS-HoU (see Table 1). Chromosomal aberration regarding the *MAT* locus induces generation of two kinds of yeast cells, *i.e.*, a- and α-type of cells. Here we described yeast cells possessing two sets of *MAT* genes (generated via LOH) as an example. Then, spontaneous or engineered formation of the a1-α2 complex suppresses the mating response by repressing expression of haploid-specific genes (*hsg*), which encode components of the mating signaling pathway. Target cells expressing the *URA3* selection marker gene can be isolated on SD – Ura plates in the absence of autopolyploidization. The target cells also express the *GFP* reporter gene. Regulation colored with cyan indicates indirect gene repression; the a1-α2 complex prevents expression of transcriptional activator α1 that is required for gene expression under the control of *P*_*STE3*_. Note that *URA3* gene is directly repressed by a transcriptional repressor in non-target cells.

As described above, the plasmid pLS-2U (constructed in trial 2) was preferred for isolation of a-type of cells, and the combination of plasmids pHS-HoU and pL3G-2α (as described in trial 3) was preferred for isolation of α-type of cells. A model that describes how our artificial gene network can function in isolation of a- or α-type of cells is shown in Figure 5.

For isolation of a-type of cells, we introduced plasmid pLS-2U into the parental strain in combination with plasmid pH2G-Pa1 (see Additional file 1: Figure S1A and B), which prevents autopolyploidization (Table 1 and Figure 5A). The a1-α2 complex represses expression of *hsg* that are required for mating signal transduction in α-type of cells generated via chromosomal aberration, as well as in parental *MAT*a/α cells. In this system, only a-type of cells can survive on SD – Ura plates, because of a-type-specific *URA3* gene expression. To confirm the mating-type of yeast cells isolated on SD – Ura plates using this method, we used a *GFP* reporter gene under the control of the a-type-specific promoter [15]. GFP-fluorescence was observed in these yeast isolates, verifying the a-type, and confirming that this system can distinguish positives from false-positives in the scheme proposed in Figure 1A.

**Table 1:** Yeast strains and plasmids used in this study

**Name**	**Description**	**Reference source**
*Yeast strains*		
BY4741	*MAT***a***his3*Δ*1 ura3*Δ*0 leu2*Δ*0 met15*Δ*0*	Brachmann *et al.*, [24]
BY4742	*MAT***α***his3*Δ*1 ura3***Δ***0 leu2*Δ*0 lys2*Δ*0*	Brachmann *et al.*, [24]
BY4743	*MAT***a***/***α***his3Δ1/his3Δ1 leu2Δ0/leu2Δ0 LYS2/lys2Δ0 met15Δ0/MET15 ura3Δ0/ura3Δ0*	Brachmann *et al.*, [24]
BY4743A	*MAT***a***his3Δ1 leu2Δ0 LYS2 MET15 ura3Δ0*	Present study
BY4743AL	*MAT***α***/***α***his3Δ1/his3Δ1 leu2Δ0/leu2Δ0 LYS2/lys2Δ0 met15Δ0/MET15 ura3Δ0/ura3Δ0*	Present study
*Plasmids*		
pRS313	Yeast expression vector; *CEN6/ARSH4* ori and *HIS3* marker	NBRP^*^
pRS315	Yeast expression vector; *CEN6/ARSH4* ori and *LEU2* marker	NBRP
pRS316	Yeast expression vector; *CEN6/ARSH4* ori and *URA3* marker	NBRP
pHY-2GA	Yeast expression vector; *2 μ* ori, *HIS3* marker and *P*_*STE2*_*-EGFP*	Fukuda *et al.*, [18]
pLY-3GC	Yeast expression vector; *2 μ* ori, *LEU2* marker and *P*_*STE3*_*-EGFP*	Fukuda *et al.*, [18]
pH2G-Pa1	*P*_*PGK1*_*-*a*1* in pHY-2GA	Fukuda *et al.*, [18]
pL3G-2α	*P*_*STE2*_*-*α*2* in pLY-3GC	Fukuda *et al.*, [18]
pHY-2U	Yeast expression vector; *2 μ* ori, *HIS3* marker and *P*_*STE2*_*-URA3*	Present study
pLY-3U	Yeast expression vector; *2 μ* ori, *LEU2* marker and *P*_*STE3*_*- URA3*	Present study
pLS-2U	*P*_*STE2*_*-URA3* in pYO315	Present study
pHS-3U	*P*_*STE3*_*-URA3* in pYO313	Present study
pHS-HoU	*P*_*HO*_*-URA3* in pYO313	Present study

^*^Resources were provided by the National Bio-Resource Project (NBRP) of the MEXT, Japan.

Next, a plasmid set composed of pHS-HoU and pL3G-2α was introduced into the parental strain for isolation of α-type of cells. pL3G-2α also provides the autopolyploidization prevention function (Table 1 and Figure 5B). The resulting a1-α2 complex represses expression of *hsg* in a-type of cells as well as in the parental *MAT*a/α cells. In this system, only α-type of cells can survive on SD – Ura plates, because of α-type-specific *URA3* gene expression. To confirm the mating-type of isolated yeast cells, we used a *GFP* reporter gene under the control of the α-type-specific promoter [18]. GFP-fluorescence was observed in these yeast isolates, verifying the α-type, and confirming the validity of the scheme proposed in Figure 1B.

### Isolation of a- and α-type of cells generated via spontaneous chromosomal aberration

**Figure 6:**
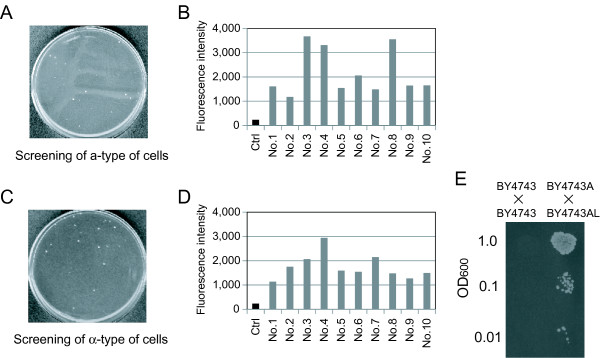
**Isolation of a- or α-type of cells generated via spontaneous chromosomal aberration. (A)** Direct images of colony formation in isolation of a-type of cells. Cell suspensions containing approximately 1 × 10^7^ yeast cells (1 mL of suspension at OD_600_ 1.0) were spread on selective solid medium (SD – Ura plate). **(B)** Fluorescent reporter assay to quantify expression of the *GFP* reporter gene in yeast transformants possessing plasmid pH2G-Pa1 (*P*_*STE2*_-*GFP*). Ctrl indicates the parental *MAT*a/α transformant (negative control). **(C)** Direct images of colony formation in isolation of α-type of cells. Cell suspensions containing approximately 1 × 10^6^ yeast cells (1 mL of suspension at OD_600_ 0.1) were spread on selective solid medium. **(D)** Fluorescent reporter assay to quantify expression of the *GFP* reporter gene in yeast transformants possessing plasmid pL3G-2α (*P*_*STE3*_-*GFP*). Ctrl indicates the parental *MAT*a/α transformant (negative control). **(E)** Mating assays to investigate the mating abilities of yeast cells. Each spot corresponds to 10 μL of suspension at the indicated OD_600_.

As shown in Figure 1A, *MAT*a/α cells possessing both plasmids pLS-2U and pH2G-Pa1 were washed and spread on SD – Ura plates after overnight cultivation with uracil. From approximately 1 × 10^7^ cells spread on the SD – Ura plate, 28 colonies were formed (Figure 6A). Ten of these colonies were randomly selected, and fluorescence intensity was measured. Values were compared to that of the parental *MAT*a/α cells (harboring pH2G-Pa1), which were used as a negative control (labeled as “Ctrl” in Figure 6B). All of the isolates derived from the SD – Ura plates exhibited higher fluorescence than Ctrl, suggesting that the proposed selection system for a-type of cells rarely produced false-positives. We selected the colony (No. 3) with the highest fluorescence intensity and performed another round of colony isolation to remove the cells of all plasmids. The resulting strain was designated BY4743A (Table 1).

Next, *MAT*a/α cells possessing both plasmids pHS-HoU and pL3G-2α were washed and spread on the SD – Ura plates after overnight cultivation with uracil, as shown in Figure 1B. From approximately 1 × 10^6^ cells spread on the SD – Ura plate, 23 colonies were formed (Figure 6C). Ten of these colonies were randomly selected, and fluorescence intensity was measured. Values were compared to that of the parental *MAT*a/α cells (harboring pL3G-2α), which were used as a negative control (labeled as “Ctrl” in Figure 6D). All of the colonies isolated on the SD – Ura plates exhibited higher fluorescence than Ctrl, suggesting that the proposed selection system for α-type of cells rarely produced false-positives. We selected the colony (No. 4) with the highest fluorescence intensity and performed another round of colony isolation to remove the cells of all plasmids. The resulting strain was designated BY4743AL (Table 1).

To verify the mating ability of BY4743A and BY4743AL, mating assays [20,21] were carried out as follows. Because BY4743A and BY4743AL have the same auxotrophy as BY4743, pH2G-Pa1 (containing *HIS3* marker) was introduced into BY4743A (a-type) and BY4743 (*MAT*a/α; parental strain), and pL3G-2α (containing *LEU2* marker) was introduced into BY4743AL (α-type) and BY4743. This allowed us to select zygotes using solid medium lacking histidine and leucine. The mating assays revealed successful mating between BY4743A and BY4743AL, but not between the two kinds of BY4743 transformants (Figure 6E). These results confirmed the validity of our proposed scheme. We believe that our approach will find application in manufacturing mating strains for yeast crossbreeding.

## Discussion

The aim of this study was to establish a versatile method, using growth-based selection, for the isolation of mating strains for yeast crossbreeding (Figure 1). Although mating strains are traditionally prepared by sporulation from *MAT*a/α yeast strains used for industrial purposes, there are numerous strains that have extreme difficulty sporulating [7]. Alternative approaches using HO endonuclease have been developed in order to provide mating strains without sporulation [18,22]. Unfortunately, however, these approaches cannot be applied to yeast strains containing the “stuck” mutation (a single base substitution) at the *MAT* locus [23]. Here we focused on chromosomal aberration such as LOH and chromosome loss, alternative processes that produce a- and α-type of cells from parental *MAT*a/α cells. Although chromosomal aberration is remarkably tolerant of slight differences in the base sequences at the *MAT* locus, spontaneous frequencies of such events are less than 1 × 10^-4^.

Hashimoto *et al.* used UV irradiation to increase the frequency of LOH [16], and Whittaker *et al.* used 12 kinds of chemicals to increase the frequency of chromosome loss [17]; however, these treatments are likely to randomly induce additional mutations and loss of other chromosomes. Because the purpose of yeast crossbreeding is to combine favorable traits of parental strains, these undesirable changes would be a concern for the resulting a- or α-type of cells. To isolate a- and α-type of cells generated via spontaneous chromosomal aberration in the absence of mutagens, we describe here the development of mating-type-dependent *URA3* gene expression systems (Figure 1).

Generally, chromosomal stability becomes diminished by an increase in ploidy (*e.g.* less stable in triploid and tetraploid than in diploid cells). According to past report, the frequency of losing chromosome VII was approximately 10,000-fold higher in tetraploid than in diploid cells of *S. cerevisiase*[15]. In principle, mating-type-dependent screening cannot exclude triploid, tetraploid and other higher polyploid cells that may emerge via chromosome aberration following autopolyploidization (mating between the a- and α-type of cells generated from the same parental strain). Although these cells would presumably be available for yeast crossbreeding, chromosome stability of the hybrids should inevitably become diminished due to unneeded increase in ploidy. Hence, we utilized artificial formation of the a1-α2 complex [18] to remove the potential risk of autopolyploidization.

To develop growth selection systems for mating strains, we initially adopted *P*_*STE2*_ (for isolation of a-type of cells) and *P*_*STE3*_ (for isolation of α-type of cells as the promoters to express the *URA3* selection marker gene. By the use of YCp-type of vector, *P*_*STE2*_-*URA3* successfully permitted only a-type of yeast cells to grow without uracil. Compared to *P*_*STE2*_, regulation of gene expression under the control of *P*_*STE3*_ was leaky, a fact that had not been noted in a previous study using the *GFP* reporter gene [18]. Although we attempted to reduce leakiness of the *URA3* gene expression by inclusion of the artificially formed a1-α2 complex (see Additional file 1: Figure S1C and D), little change was seen in the OD_600_ value of *MAT*a and *MAT*a/α cells after 24 h cultivation (see Additional file 1: Figure S1D; compare to Figure 3D).

The difference in leakiness between *P*_*STE2*_ and *P*_*STE3*_ may reflect the distinct regulatory mechanisms controlling *asg* and *αsg* promoters. In *MAT*α and *MAT*a*/*α cells, expression of *asg* is suppressed by the α2 repressor encoded by the *MAT*α allele [24]. The *MAT*α allele also encodes the α1 activator, which facilitates the expression of *αsg*. Whereas *MAT*a cells rarely express *αsg* due to absence of the α1-coding gene within the *MAT* locus, *MAT*a/α cells are unlikely to express *αsg* because the α1-coding gene is repressed by the a1-α2 complex (a1 is encoded by the *MAT*a allele) [19]. Unlike promoters of *asg*, there is no component that can directly repress promoters of *αsg*, which might permit a low level of basal (leaky) expression. In the present work, we succeeded in tight α-type-specific *URA3* gene expression by utilizing *P*_*HO*_, a promoter of *hsg* that is directly repressed by the a1-α2 complex (Figure 4).

While use of auxotrophic markers has an advantage in transformation efficiencies, the utility of them is limited within laboratory strains or genetically modified strains because industrial yeast strains usually do not have auxotrophic mutations such as *ura3*, *his3* and *leu2*. However, in principle, our system can adopt drug-resistance markers which are available for industrial yeast strains. Instead of the *URA3* marker, we attempted to use *kanMX4* marker (see Additional file 2) that gives resistance for geneticin (G418), and succeeded in growing only target cells in cultivation medium containing G418 (see Additional file 3: Figure S2).

To evaluate the availability of the established growth selection systems, we manufactured a- and α-type of cells as shown in Figure 1. The appearance frequencies of a- and α-type of cells were 2.8 × 10^-6^ and 2.3 × 10^-5^, respectively. Because the frequency of chromosomal aberration generally varies by yeast strain, genetic modifications might have some effect on the frequency of chromosomal aberration in the parental BY4743 strain. Next, we verified the mating-type of colonies isolated on SD – Ura plates using *GFP* reporter gene expression. All colonies had the expected mating-type, suggesting the utility of isolating a- and α-type of cells via the constructed growth-based selection systems.

We performed another round of colony isolation to remove all plasmids from yeast cells, yielding BY4743A and BY4743AL strains, respectively. To identify the event generating BY4743A or BY4743AL strain, we carried out quantitative analysis for the number of chromosome III (containing *MAT* genes) using real-time PCR (see Additional file 4: Figure S3). BY4743A strain has only 1 set of chromosome III, suggesting that it was generated via chromosome loss. On the other hand, BY4743AL strain has 2 sets of chromosome III, suggesting that it was generated via LOH.

Furthermore, to confirm the stability of their mating ability, we carried out mating assays after serial passage in culture (see Additional file 5: Figure S4). Even after three passages, BY4743A and BY4743AL continued to exhibit equivalent levels of mating. We expect that the mating abilities of the generated a- and α-type of cells will remain extremely stable, given that additional chromosomal rearrangements are unlikely to occur at the *MAT* loci.

## Conclusion

We have established a new approach to manufacture a- and α-type of cells from parental *MAT*a/α cells using growth selection systems. Use of spontaneous chromosomal aberration is quite beneficial in acquisition of mating strains inheriting desirable properties of industrially-used strains. We showed that yeast strains generated via spontaneous chromosomal aberration have stable mating ability, providing hybrid strains for use in yeast crossbreeding. Use of our method should help promote further advances of yeast-based biosynthesis approaches and in other experimental areas of research such as quantitative trait locus analysis and genome-wide association studies that permit the linking of phenotypic traits and genotypic data.

## Methods

### Strains and media

Detailed information about *Saccharomyces cerevisiae* strains BY4741, BY4742, and BY4743 [25], as well as other strains used in this study, is shown in Table 1. Yeast cells were grown in YPD medium (1% yeast extract, 2% peptone and 2% glucose) or in SD medium (0.67% yeast nitrogen base without amino acids (Becton Dickinson and Company, Franklin Lakes, NJ, USA) and 2% glucose). A final concentration of 2% agar was added to make solid media.

### Construction of plasmids

The sequences of oligonucleotides used in this study are shown in Table 2. The plasmids shown in Table 1 were made as follows. Using pRS316 (provided by the National BioResource Project (NBRP) of the MEXT, Japan) as a template, the *URA3* gene was amplified with oligonucleotide pair o1 and o2, and inserted in place of the *EGFP* at the *Not*I-*Bam*HI sites of pHY-2GA [18], yielding a YEp-type plasmid designated pHY-2U. Similarly, using pRS316 as a template, the *URA3* gene was amplified with oligonucleotide pair o3 and o2, and inserted in place of the *EGFP* at the *Not*I-*Bam*HI sites of pLY-3GC [18], yielding a YEp-type plasmid designated pLY-3U.

**Table 2:** Sequences of oligonucleotides used to construct plasmids

**Number**	**Sequence**
1	5′-gaatcaaaaGCGGCCGCatgtcgaaagctacatat-3′
2	5′-ccccagtttgGGATCCttagttttgctggccgcat-3′
3	5′-aaaattttcGCGGCCGCatgtcgaaagctacatat-3′
4	5′-aattggagctccaCCGCGG-3′
5	5′-cgggccccccCTCGAG-3′
6	5′-aattggagctccaCCGCGGcatttttgtttcttttggac-3′
7	5′-tttcgacatGCGGCCGCtttaaagtatagatagaa-3′

The YCp-type plasmids used to express the *URA3* gene were constructed as follows. DNA fragments containing *P*_*STE2*_-*URA3* were amplified from pHY-2U using oligonucleotide pair o4 and o5, and inserted into the *Sac*II-*Xho*I sites of pRS315 (provided by NBRP), yielding the plasmid pLS-2U. DNA fragments containing *P*_*STE3*_-*URA3* were amplified from pLY-3U using oligonucleotide pair o4 and o5, and inserted into the *Sac*II-*Xho*I sites of pRS313 (provided by NBRP), yielding the plasmid pHS-3U. The haploid-type-specific promoter, *P*_*HO*_, was amplified using oligonucleotide pair o6 and o7 from genomic DNA derived from strain BY4741, and inserted in place of *P*_*STE3*_ at the *Sac*II-*Not*I sites of pHS-3U, yielding the plasmid pHS-HoU. Each plasmid was introduced into yeast cells using the lithium acetate method [26].

### Investigation of cell growth characteristics

Each yeast transformant was grown in 500 μL of SD medium without or with 20 mg/L uracil at 30°C, setting initial optical density at 600 nm (OD_600_) at 0.03. The OD_600_ values of cultures were monitored using a UV/visible spectrophotometer (Ultrospec 3100 pro; GE Healthcare Japan Corporation, Tokyo, Japan).

### Fluorescent reporter assay

The *EGFP* gene was used as a fluorescent reporter to indicate mating type. Reporter-containing cells were incubated at 30°C for 18 h, harvested and washed with distilled water. The cells then were resuspended in 100 μL of distilled water to an OD_600_ of 5.0. GFP fluorescence intensities were measured using an Infinite 200 fluorescence microplate reader (Tecan Japan Co., Ltd., Kawasaki, Japan). For detection of the GFP signal, the excitation wavelength was set at 485 nm with a bandwidth of 20 nm, and the emission wavelength was set at 535 nm with a bandwidth of 25 nm. The gain was set at 50.

### Mating assay

Evaluation of mating ability was performed as follows. Yeast diploid cells were cultivated with the mating partner in 1 ml of YPD medium at 30°C for 1.5 h, with an initial OD_600_ of 0.1. After cultivation, yeast cells were harvested, washed, and resuspended in distilled water. Starting from an initial OD_600_ of 1, 0.1, or 0.01, 10 μl of cell suspensions were spotted on SD solid medium without the appropriate amino acids for growth selection of zygotes. After incubation at 30°C for 2 days, the image data was recorded for colonies on solid medium.

### Isolation of yeast cells with target mating-type

Parental *MAT*a/α cells were grown in 500 μL of SD media with 20 mg/L uracil at 30°C for 18 h, and then harvested and washed with distilled water. The cells then were resuspended in distilled water. Cell suspensions were spread on SD – Ura plates.

## Abbreviations

EGFP: Enhanced green fluorescent protein; LOH: Loss of heterozygosity; MAT locus: Mating-type locus; hsg: Haploid-specific genes; asg: a-type-specific genes; αsg: α-type-specific genes.

## Competing interests

We declare that all authors are inventors on a pending patent using aspects of this system.

## Authors’ contributions

NF designed the study, conducted experiments, analyzed data, and co-wrote the manuscript. SH analyzed data and co-wrote the manuscript. Both authors read and approved the final manuscript.

## Supplementary Material

Additional file 1: Figure S1 Alternative growth selection systems for isolation of a-type or α-type yeast cells by formation of the a1-α2 complex. (A) Plasmids used for a-type-specific *URA3* gene expression. The plasmid pLS-2U was used in combination with pH2G-Pa1, which suppresses the mating ability of α-type cells. (B) The OD_600_ values of cultures of double transformants (harboring both plasmids pLS-2U and pH2G-Pa1) at 24 h cultivation. Black bars indicate cultivation with uracil, and gray bars indicate cultivation without uracil. (C) Plasmids used for α-type-specific *URA3* gene expression. The plasmid pHS-3U was used in combination with pL3G-2α, which is required for suppressing the mating ability of a-type cells. (D) The OD_600_ values of cultures of double transformants (harboring both plasmids pHS-3U and pL3G-2α) at 24 h cultivation. Black bars indicate cultivation with uracil, and gray bars indicate cultivation without uracil. Click here for file

Additional file 2 **Supporting information for Materials and Methods. Table S1.** Sequences of oligonucleotides used to construct plasmids. Click here for file

Additional file 3: Figure S2 Growth of yeast transformants harboring *kanMX4* selection marker gene. (A) Plasmid map of pLS-2 K containing *CEN6/ARSH4* origin of replication (providing cellular retention of single-copy plasmids) and *P*_*STE2*_-*kanMX4* construct (activated in a-type yeast cells). (B) The growth curves of pLS-2 K transformants. Closed symbols indicate cultivation without G418, and open symbols indicate cultivation with G418. (C) Plasmid map of pHS-HoK-2α containing *CEN6/ARSH4* origin of replication and *P*_*HO*_-*kanMX4* construct combined with *P*_*STE2*_-α2 construct (activated in α-type yeast cells). (D) The growth curves of pHS-HoK-2α transformants. Symbols are as in B. Click here for file

Additional file 4: Figure S3 Ploidy analysis using real-time PCR. The normalized copy number of the *PGK1* gene is an indicator of ploidy (A) for BY4743A and (B) for BY4743AL strains. Standard deviations of three replicates are presented. Click here for file

Additional file 5: Figure S4 Investigation of stability of the mating abilities of yeast cells after serial passage of cultures. Up to three serial passages were carried out, and then the resulting BY4743, BY4743A, and BY4743AL transformants were used for mating assays. Click here for file
