# Supplementary figures and images for: Development of growth selection systems to isolate a-type or α-type of yeast cells spontaneously emerging from MATa/α diploids
Source: J Biol Eng. 2013 Nov 21;7:27. doi: 10.1186/1754-1611-7-27 (PMC3923440; doi:10.1186/1754-1611-7-27)

A

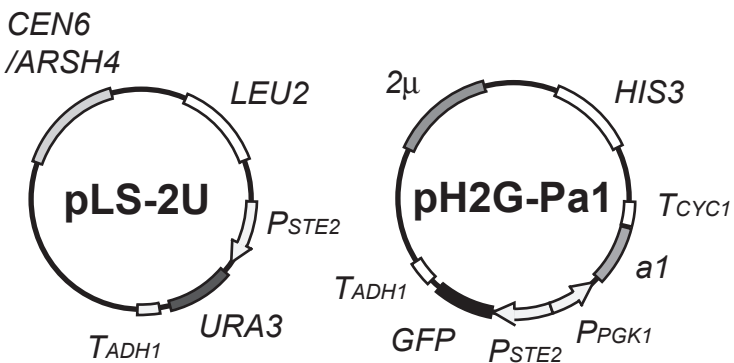

C

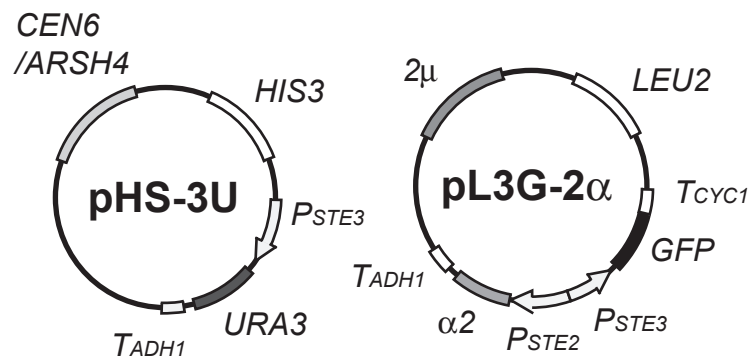

B

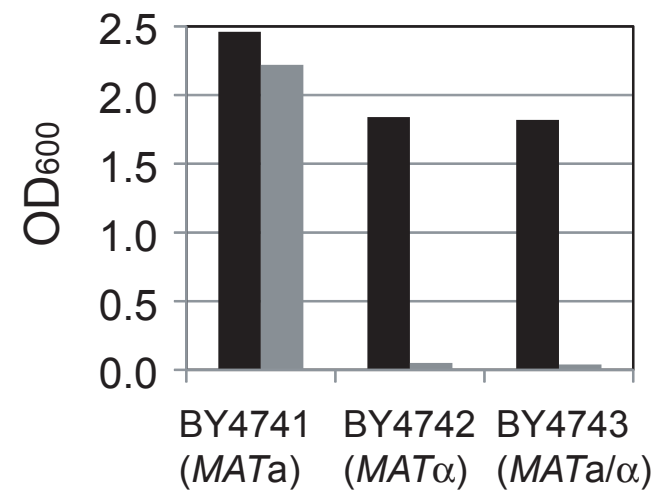

D

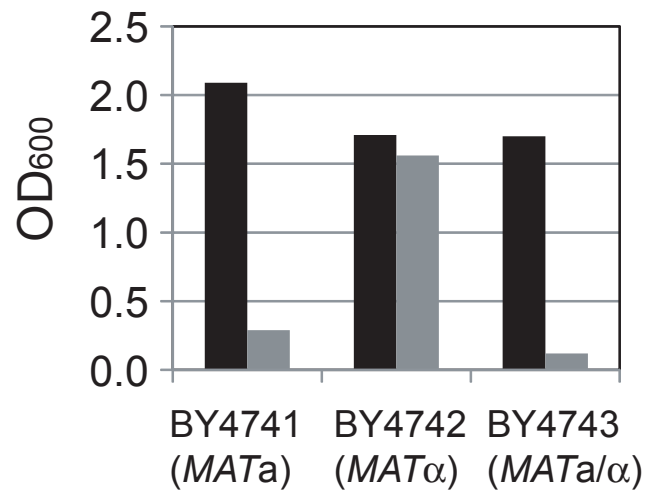

Supplement: Additional file 1: Figure S1 — Alternative growth selection systems for isolation of a-type or α-type yeast cells by formation of the a1-α2 complex. (A) Plasmids used for a-type-specific URA3 gene expression. The plasmid pLS-2U was used in combination with pH2G-Pa1, which suppresses the mating ability of α-type cells. (B) The OD600 values of cultures of double transformants (harboring both plasmids pLS-2U and pH2G-Pa1) at 24 h cultivation. Black bars indicate cultivation with uracil, and gray bars indicate cultivation without uracil. (C) Plasmids used for α-type-specific URA3 gene expression. The plasmid pHS-3U was used in combination with pL3G-2α, which is required for suppressing the mating ability of a-type cells. (D) The OD600 values of cultures of double transformants (harboring both plasmids pHS-3U and pL3G-2α) at 24 h cultivation. Black bars indicate cultivation with uracil, and gray bars indicate cultivation without uracil. [file 1754-1611-7-27-S1.pdf]

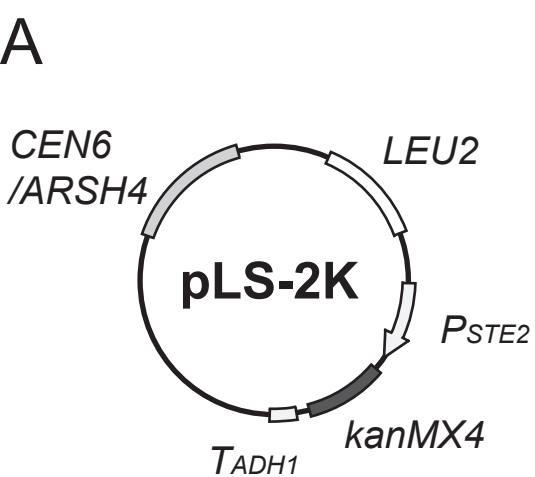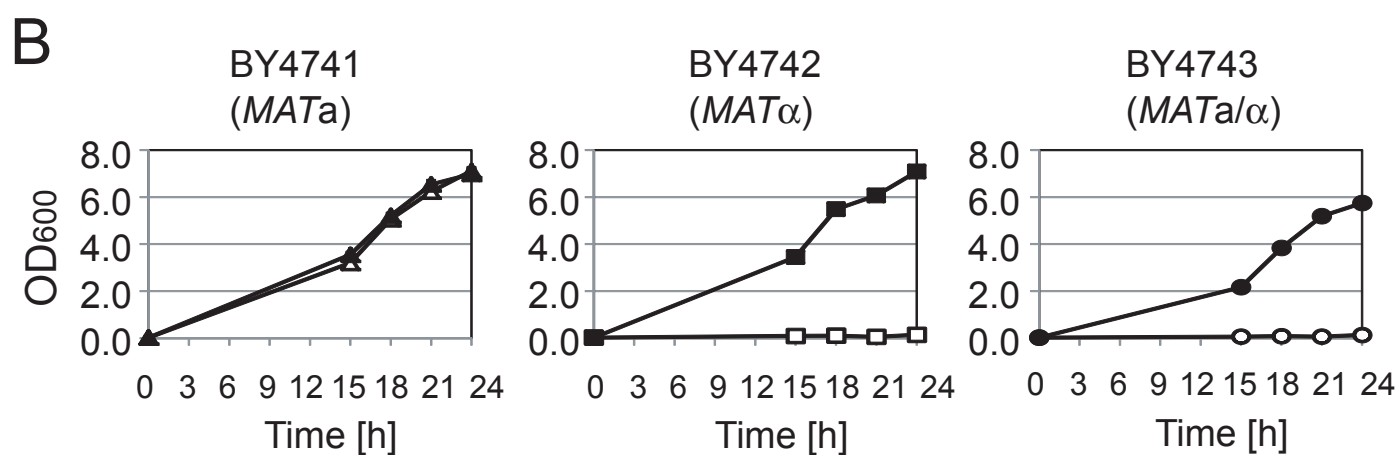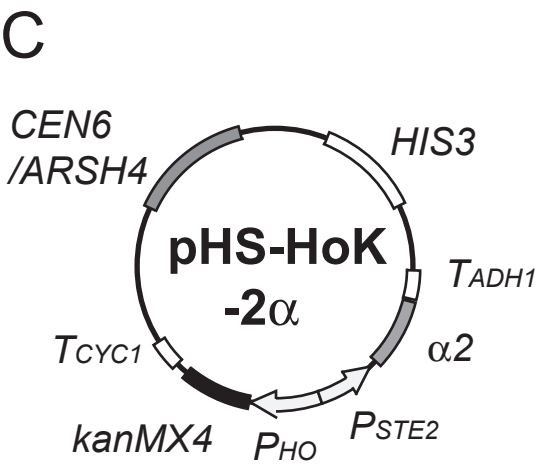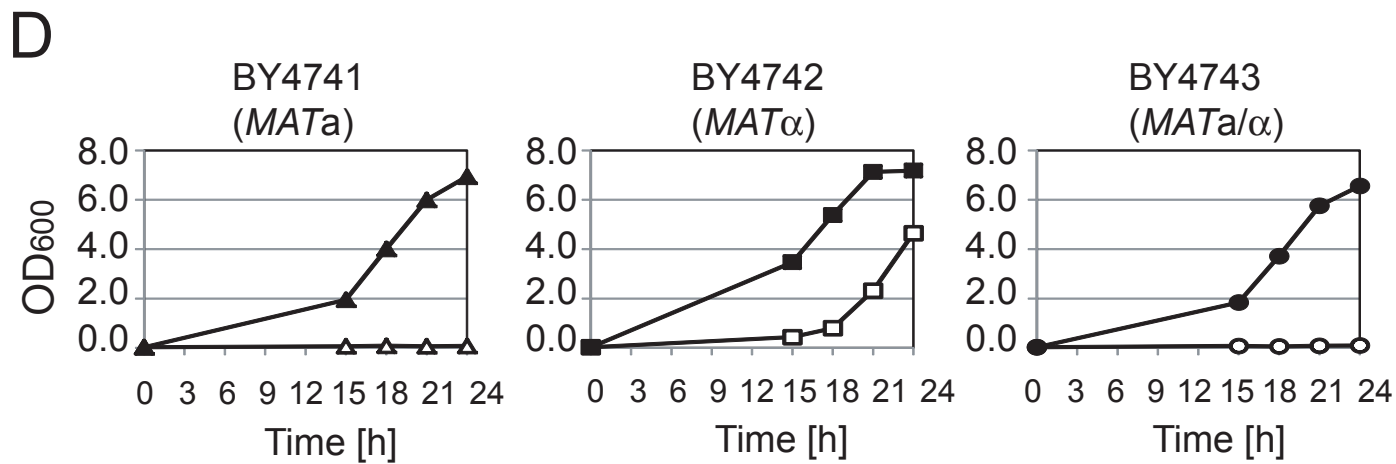

Supplement: Additional file 3: Figure S2 — Growth of yeast transformants harboring kanMX4 selection marker gene. (A) Plasmid map of pLS-2 K containing CEN6/ARSH4 origin of replication (providing cellular retention of single-copy plasmids) and P STE2 -kanMX4 construct (activated in a-type yeast cells). (B) The growth curves of pLS-2 K transformants. Closed symbols indicate cultivation without G418, and open symbols indicate cultivation with G418. (C) Plasmid map of pHS-HoK-2α containing CEN6/ARSH4 origin of replication and P HO -kanMX4 construct combined with P STE2 -α2 construct (activated in α-type yeast cells). (D) The growth curves of pHS-HoK-2α transformants. Symbols are as in B. [file 1754-1611-7-27-S3.pdf]

BY4743

×

BY4743

BY4743A

×

BY4743AL

BY4743A

×

BY4743AL

BY4743A

×

BY4743AL

OD<sub>600</sub>

1.0

0.1

0.01

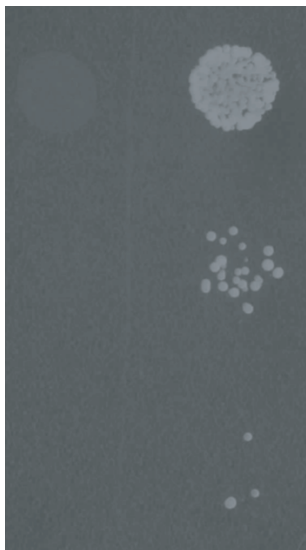

Passage 1

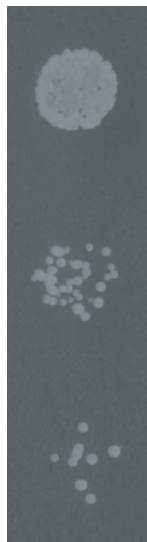

Passage 2

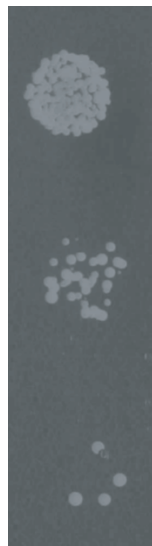

Passage 3

Supplement: Additional file 5: Figure S4 — Investigation of stability of the mating abilities of yeast cells after serial passage of cultures. Up to three serial passages were carried out, and then the resulting BY4743, BY4743A, and BY4743AL transformants were used for mating assays. [file 1754-1611-7-27-S5.pdf]
