# Supplementary material for: Development of growth selection systems to isolate a-type or α-type of yeast cells spontaneously emerging from MATa/α diploids
Source: J Biol Eng. 2013 Nov 21;7:27. doi: 10.1186/1754-1611-7-27 (PMC3923440; doi:10.1186/1754-1611-7-27)
Supplement: Additional file 2 — Supporting information for Materials and Methods. Table S1. Sequences of oligonucleotides used to construct plasmids. [file 1754-1611-7-27-S2.doc]

**Supporting Information**

**Development of Growth Selection Systems to Isolate a-type or α-type of Yeast Cells Spontaneously Emerging from *MAT*a/α diploids**

Nobuo Fukuda and Shinya Honda*****

Biomedical Research Institute, National Institute of Advanced Industrial Science and Technology (AIST), Higashi, Tsukuba, Ibaraki 305-8566, Japan.

*Corresponding author

Fax: +81 29 861 6194

Tel: +81 29 861 9444

E-mail: s.honda@aist.go.jp

**Doc. S1**

**Supporting information for Materials and Methods**

**Construction of plasmids using *kanMX4* marker.** The oligonucleotides used in this study are summarized in Table S1. Using pK6 [1] as a template, the *kanMX4* gene was amplified with oligonucleotide pair o1 and o2, and inserted in place of the *URA3* at the *Not*I-*Bam*HI sites of pLS-2U, yielding plasmid pLS-2K. Similarly, pK6 as a template, the *kanMX4* gene was amplified with oligonucleotide pair o3 and o2, and inserted in place of the *URA3* at the *Not*I-*Bam*HI sites of pHS-3U, yielding plasmid pHS-3K.

Promoter of *HO* gene (*P_HO_*) was amplified from pHS-HoU with oligonucleotide pair o4 and o5, and DNA fragments containing *P_STE2_*-*α2*-*T_ADH1_* were amplified from pH2Y-α2 [1] with oligonucleotide pair o6 and o7. Using In-Fusion Cloning Kit (Takara Bio, Otsu, Japan), the amplified two kinds of DNA fragments were inserted in place of *P_STE3_* at the *Sac*I-*Sac*II sites of pHS-3K. The yielding plasmid was termed as pHS-HoK-2α.

**Investigation of cell growth characteristics.** Each yeast transformant was grown in 500 μL of YPD medium without or with 500 μg/L G418 at 30°C, setting initial optical density at 600 nm (OD_600_) at 0.03. The OD_600_ values of cultures were monitored using a UV/visible spectrophotometer (Ultrospec 3100 pro; GE Healthcare Japan Corporation, Tokyo, Japan).

**Ploidy analysis using real-time PCR.** The generated strains in the current study were mated with the opposite mating-type strains, MCF4741 (*MAT*a) [1] or HR42-11T (*MAT*α) [1]. These strains have one copy of the *kanMX4* gene integrated into the yeast chromosome DNA, which serves as a reference for ploidy analysis. Template genomic DNA was isolated from yeast cells cultivated in YPD medium at 30 °C for 18 h. Quantitative real-time PCR was performed using an ABI PRISM 7900HT Sequence Detection System (Applied Biosystems, Foster City, CA) with Thunderbird SYBR qPCR Mix (Toyobo, Osaka, Japan). Two sets of PCR primers, o8 and o9, and o10 and o11, were used to detect the *kanMX4* and *PGK1* genes respectively. Because one copy of the *PGK1* gene exists in one set of chromosomal DNA (on chromosome III), the normalized copy number of *PGK1* can be calculated using the standard curve method together with *kanMX4* as the reference for 1 copy/cell, such that the *PGK1*/*kanMX4* ratio is an indicator of ploidy. The chromosome ploidy of the generated strains was estimated by subtracting 1 (corresponding to the chromosome ploidy of the mating partners) from that of the zygotes.

**References**

[1] Fukuda, N., Matsukura, S. and Honda, S. (2013). Artificial conversion of the mating-type of *Saccharomyces cerevisiae* without autopolyploidization. ACS Synth Biol., in press.

**Table S1.** Sequences of oligonucleotides used to construct plasmids.

Number Sequence

1 5’-GAATCAAAAgcggccgcATGGGTAAGGAAAAGACT-3’

2 5’-CCCCAGTTTGggatccTTAGAAAAACTCATCGAGC-3’

3 5’-AAAATTTTCgcggccgcATGGGTAAGGAAAAGACT-3’

4 5’-AATTGGAGCTCCAccgcggCATTTTTGTTTCTTTTGGA-3’

5 5’- CTTACCCATgcggccgcTTTAAAGTATAGATAGAA-3’

6 5’-CGGTGgagctcCAATTATCCAATATCACCTGACC-3’

7 5’-TATAGGGCGAATTGgagctcCGGGCCCCCCctcgag-3’

8 5’-AATCAGGTGCGACAATCTATCGA-3’

9 5’-CAACGCTACCTTTGCCATGTT-3’

10 5’-GCCCCAGGTTCCGTTATTTT-3’

11 5’-ACCTTTTGACCATCGACCTTTC-3’

**Supplementary Figure Legends**

**Figure S1. Alternative growth selection systems for isolation of a-type or α-type yeast cells by formation of the a1-α2 complex.** (A) Plasmids used for a-type-specific *URA3* gene expression. The plasmid pLS-2U was used in combination with pH2G-Pa1, which suppresses the mating ability of α-type cells. (B) The OD_600_ values of cultures of double transformants (harboring both plasmids pLS-2U and pH2G-Pa1) at 24 h cultivation. Black bars indicate cultivation with uracil, and gray bars indicate cultivation without uracil. (C) Plasmids used for α-type-specific *URA3* gene expression. The plasmid pHS-3U was used in combination with pL3G-2α, which is required for suppressing the mating ability of a-type cells. (D) The OD_600_ values of cultures of double transformants (harboring both plasmids pHS-3U and pL3G-2α) at 24 h cultivation. Black bars indicate cultivation with uracil, and gray bars indicate cultivation without uracil.

**Figure S2. Growth of yeast transformants harboring *kanMX4* selection marker gene.** (A) Plasmid map of pLS-2K containing *CEN6/ARSH4* origin of replication (providing cellular retention of single-copy plasmids) and *P_STE2_*-*kanMX4* construct (activated in a-type yeast cells). (B) The growth curves of pLS-2K transformants. Closed symbols indicate cultivation without G418, and open symbols indicate cultivation with G418. (C) Plasmid map of pHS-HoK-2α containing *CEN6/ARSH4* origin of replication and *P_HO_*-*kanMX4* construct combined with *P_STE2_*-α2 construct (activated in α-type yeast cells). (D) The growth curves of pHS-HoK-2α transformants. Symbols are as in B.

**Figure S3. Ploidy analysis using real-time PCR.** The normalized copy number of the *PGK1* gene is an indicator of ploidy (A) for BY4743 and (B) for BY4743AL strains. Standard deviations of three replicates are presented.

**Figure S4. Investigation of stability of the mating abilities of yeast cells after serial passage of cultures.** Up to three serial passages were carried out, and then the resulting BY4743, BY4743A, and BY4743AL transformants were used for mating assays.
