# Supplementary material for: Development of growth selection systems to isolate a-type or α-type of yeast cells spontaneously emerging from MATa/α diploids
Source: J Biol Eng. 2013 Nov 21;7:27. doi: 10.1186/1754-1611-7-27 (PMC3923440; doi:10.1186/1754-1611-7-27)
Supplement: Additional file 4: Figure S3 — Ploidy analysis using real-time PCR. The normalized copy number of the PGK1 gene is an indicator of ploidy (A) for BY4743A and (B) for BY4743AL strains. Standard deviations of three replicates are presented. [file 1754-1611-7-27-S4.pdf]

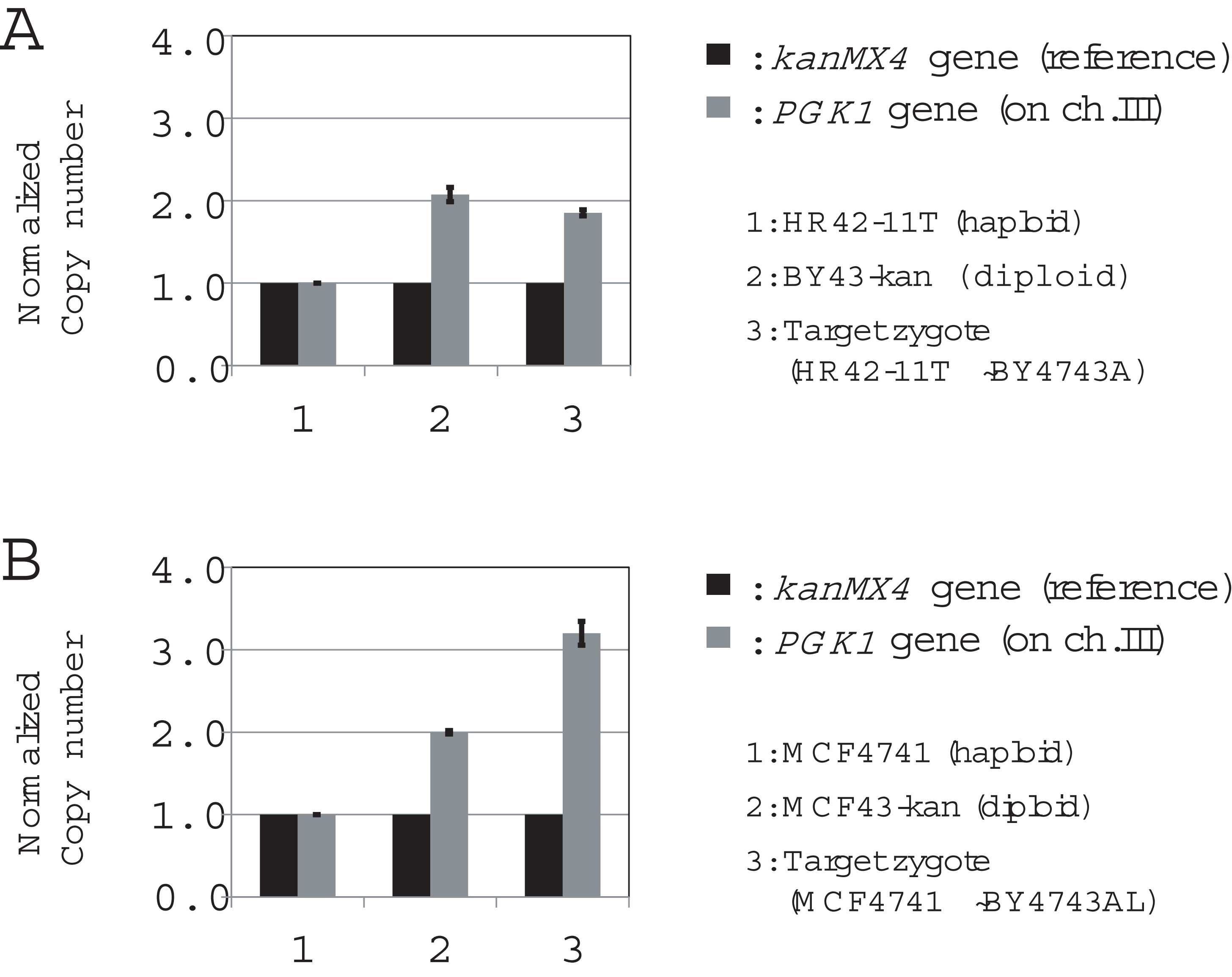
A
Normalized Copy number
4.0
3.0
2.0
1.0
0.0
1
2
3
: kanMX4 gene (reference)
: PGK1 gene (on ch.III)
1: HR42-11T (haploid)
2: BY43-kan (diploid)
3: Target zygote (HR42-11T × BY4743A)
B
Normalized Copy number
4.0
3.0
2.0
1.0
0.0
1
2
3
: kanMX4 gene (reference)
: PGK1 gene (on ch.III)
1: MCF4741 (haploid)
2: MCF43-kan (diploid)
3: Target zygote (MCF4741 × BY4743AL)
